# Country data on AMR in Mexico in the context of community-acquired respiratory tract infections: links between antibiotic susceptibility, local and international antibiotic prescribing guidelines, access to medicine and clinical outcome

**DOI:** 10.1093/jac/dkac216

**Published:** 2022-09-06

**Authors:** Didem Torumkuney, Carlos de la Torre, Karen Langfeld, Norma Patricia Lopez-Turrent, Cristiana Ossaille Beltrame

**Affiliations:** GlaxoSmithKline, 980 Great West Road, Brentford, Middlesex TW8 9GS, UK; Department of Paediatric Otolaryngology, Hospital Infantil de México, Federico Gómez, Mexico City, Mexico; GlaxoSmithKline, 980 Great West Road, Brentford, Middlesex TW8 9GS, UK; GlaxoSmithKline, Torre Mítikah Piso 19 y 20. Circuito Interior Avenida Río Churubusco 601, Col. Xoco, Benito Juárez, C.P. 03330 Mexico City, Mexico; GlaxoSmithKline, Estrada dos Bandeirantes, 8464, Jacarepaguá, 22783-110 Rio de Janeiro, Brazil

## Abstract

**Background:**

Antimicrobial resistance (AMR) is one of the biggest threats to global public health. Selection of resistant bacteria is driven by inappropriate use of antibiotics, amongst other factors. COVID-19 may have exacerbated AMR due to unnecessary antibiotic prescribing. Country-level knowledge is needed to understand options for action.

**Objectives:**

To review AMR in Mexico and initiatives addressing it. Identifying any areas where more information is required will provide a call to action to minimize any further rises in AMR and to improve patient outcomes.

**Methods:**

National AMR initiatives in Mexico, antibiotic use and prescribing, and availability of susceptibility data, particularly the key community-acquired respiratory tract infection (CA-RTI) pathogens *Streptococcus pneumoniae* and *Haemophilus influenzae*, were identified. National and international antibiotic prescribing guidelines commonly used in Mexico for specific CA-RTIs (community-acquired pneumonia, acute otitis media and acute bacterial rhinosinusitis) were also reviewed, along with local antibiotic availability. Insights from a local clinician were sought to contextualize this information.

**Conclusions:**

The Mexican national AMR strategy was published in 2018. This comprised similar objectives to the Global Action Plan from the World Health Assembly (2015) and was compulsory, requiring full compliance from members of the National Health System. Historically, antibiotic consumption in Mexico has been high, however, between 2000 and 2015, consumption fell, in sharp contrast to the majority of countries. Mexico lacks a national surveillance network for AMR, however there are several ongoing global surveillance studies providing local antibiotic susceptibility data. International and local antibiotic prescribing guidelines for CA-RTIs are used. A more standardized inclusive approach in developing local guidelines, using up-to-date local surveillance data of isolates from community-acquired infections, could make guideline use more locally relevant. This would pave the way for a higher level of appropriate antibiotic prescribing and improved adherence. This would, in turn, potentially limit AMR development in Mexico and improve patient outcomes.

## Introduction

Antimicrobial resistance (AMR) is one of the biggest threats to public health throughout the world^1^ as described in the introductory paper of this Supplement.^2^ The WHO states that ‘the world urgently needs to change the way it prescribes and uses antibiotics. Even if new medicines are developed, without behaviour change, antibiotic resistance will remain a major threat’.^3^ The first paper in this Supplement included details about the multiple factors which can drive a rise in AMR along with the global initiatives that are in place to address this threat.^2^ Each country and/or region must also play their part through local initiatives.

In order to identify how AMR can be addressed in Mexico in the future, it is necessary to review what is happening now. In this paper, we present the current situation in Mexico, determined by using published information (from searching PubMed, Google Scholar and other internet platforms) to ascertain any national initiatives to address AMR, antibiotic use and prescribing, and availability of susceptibility data, in particular for the key community-acquired respiratory tract infection (CA-RTI) pathogens *Streptococcus pneumoniae* and *Haemophilus influenzae*. National and international antibiotic prescribing guidelines for CA-RTIs, specifically community-acquired pneumonia (CAP), acute otitis media (AOM) and acute bacterial rhinosinusitis (ABRS), commonly used by healthcare professionals in Mexico were also reviewed, along with how these link to local antibiotic availability. Insights from a clinician in Mexico were sought to contextualize this information. In addition, we aimed to identify areas where more information is required and present a call to action to improve clinical outcomes for patients and to minimize further rises in AMR within Mexico.

## Action Plans

Following the formulation by the World Health Assembly in 2015 of a Global Action Plan (GAP) for AMR^4^ many countries began to develop their own National Action Plan (NAP). In 2018, the Mexican government published an agreement which announced that the National Strategy of Action Against Resistance to Antimicrobials was compulsory and required full compliance from members of the National Health System.^5^ The National Strategy is a comprehensive plan which has similar objectives to the GAP, namely to improve awareness of AMR through effective communication, to strengthen knowledge of AMR in the context of both humans and animals through study of surveillance and epidemiology, to reduce the incidence of infections in both humans and animals through preventative measures, to use antimicrobials rationally in both humans and animals and to develop an economic assessment of the problem in Mexico to ensure sustainable investment. Recent analysis by the WHO presented on their website showed that in 2020–21, the plan in Mexico was at the implementation stage.^6^

## Antibiotic resistance

A recent review summarizes the progression of AMR in Mexico over a 10 year period (2009–18) through a retrospective analysis of both Gram-positive and Gram-negative bacteria by including data from 22 of the 32 Mexican states.^7^ A range of bacteria were investigated including *S. pneumoniae* isolates from respiratory tract infections (RTIs) where a decrease in resistance was observed against penicillin, cefotaxime, erythromycin and trimethoprim/sulfamethoxazole. Trends in antibiotic susceptibilities of bacteria tested were variable; for some bacteria, resistance to some antibiotics increased over the period of study. This highlights the need for regular monitoring of antibiotic susceptibilities. The study provided valuable information as it covered a wide geographic area with participation from multiple medical centres, however, the antibiotic susceptibility testing methodology was not standardized, and data were incomplete in some areas. This investigation had been preceded by a similar ‘snapshot’ study which investigated susceptibilities in 47 centres for a 6 month period. *S. pneumoniae* and *H. influenzae* were not included due to lack of information, but the authors particularly noted the multidrug resistance of *Acinetobacter* spp., *Klebsiella* spp. and *Escherichia coli* and the carbapenem resistance in specific groups of Enterobacterales. The main limitation was its duration (only 6 months), however, it reflects important information gathered from 20 Mexican states that will allow the definition of strategies for the control of resistance.^8^

## Antibiotic prescribing and usage

Historically, antibiotic consumption in Mexico has been high. In 1997, Mexico was the leading country in Latin America for antibiotic clinical use but, by 2007, Mexico was in fourth place, behind Argentina, Venezuela and Peru.^9^ This has been corroborated by a global study which showed that, unlike most of the countries throughout the world, between 2000 and 2015 the antibiotic consumption rate in Mexico fell by between 10 and 15 defined daily doses (DDD) per 1000 inhabitants per day, contrasting with the majority of countries (where consumption rose, some by as much as 25–30 DDD per 1000 inhabitants per day). This resulted in an antibiotic consumption rate for Mexico for 2015 in DDDs per 1000 inhabitants per day that placed Mexico 73rd out of the 76 countries included in the study.^10^ This reduction in antibiotic consumption could be viewed as a promising trend towards rational prescription practices or, according to some authors, could alternatively be due to a decrease in economic growth during the last three decades and inequality of income leading to the reduced possibility of purchasing antibiotics, and hence reduced consumption.^11^

Until 2010, over-the-counter (OTC) sale of antibiotics was allowable in Mexico. A systematic global study showed that pre-2010, 18% of antibiotics used in Mexico were obtained without a prescription^12^ but in that year, Mexico (and Brazil), implemented a policy enforcing the prohibition of all systemic antibiotic sales without prescription.^13^ Analysis of the effect of this prohibition concluded that the trend in decreased antibiotic use in Mexico was boosted, in particular with the reduction in use of penicillins and fluoroquinolones, which is of importance since the penicillin antibiotics had been reported to be the most common group for self-medication in Mexico.

## Surveillance

### National surveillance studies

Mexico lacks a national network for monitoring antibiotic resistance, so available data comprise local reports without uniform methodology.^11^ In 2018, a non-governmental initiative launched the Network for Research and Surveillance of Drug Resistance [Red Temática de Investigación y Vigilancia de la Farmacorresistencia (INVIFAR)] in Mexico to include the surveillance of resistance in common pathogens such as *Enterococcus* spp., *Staphylococcus aureus*, *E. coli*, *Klebsiella* spp. and in collaboration with SIREVA/GIVEBPVac (Grupo Interinstitucional para la Vigilancia de Enfermedades Bacterianas Prevenibles por Vacunación), *S. pneumoniae* but the number of isolates has been low.^7^

Recent data (2019) on the susceptibility of *S. pneumoniae* in Mexico stratified by patient age and sex are available from GIVEBPCVac.^14^ Two-hundred and fourteen isolates were from all age groups [129 from pneumonia (60.3%), 16 from meningitis (7.5%), 49 from bacteraemia (22.9%) and 20 from other diseases (9.3%)]. The proportion of non-meningitis isolates susceptible to penicillin from patients of all age groups was 68.0% (≤2.0 mg/L); 17.8% were intermediately resistant (4.0 mg/L) and 14.2% were resistant (≥8.0 mg/L). Among 213 isolates, susceptibility to erythromycin (from all age groups) was 49.3% (105/213).

### Global surveillance studies

Several ongoing global surveillance studies provide antibiotic susceptibility data in Mexico or will provide valuable data in the future.

Clinical breakpoints in surveillance studies and antibiotic susceptibility testing are cut-off MIC values used to classify microorganisms into the clinical categories susceptible (S), intermediate (I) and resistant (R), in order to assist in the prediction of the clinical success or failure of a specific antibiotic.^15^ Two international organizations define breakpoint values: CLSI and EUCAST. Due to variation in criteria for their definition, there are some differences between CLSI and EUCAST in the clinical breakpoint values for certain bacteria for some antibiotics and this can impact the susceptibility interpretation of clinical isolates.^16^ EUCAST breakpoints are dose-specific and use the EMA-approved doses that are included in the Summary of Product Characteristics of an antibiotic. This means that by application of breakpoints for higher doses, the effect of using a raised dose on the clinical efficacy of a particular antibiotic can be predicted. Currently, most clinical microbiology laboratories in Mexico use CLSI breakpoints, however the international application of the EUCAST breakpoints is expanding^17^ so it is possible that dose-specific breakpoints could at some time also be applied in Mexico, as seen in Brazil. The EUCAST dose-specific breakpoints can also be used retrospectively to calculate the susceptibility of previously collected isolates to show the susceptibility levels that would have been achieved at higher doses.

Use of the EUCAST dose-specific breakpoints shows the effect of increasing the antibiotic dose on the susceptibility of a pathogen, providing additional information so the prescriber can decide if a higher dose would be of benefit. For example, *S. pneumoniae,* the most commonly isolated respiratory pathogen^18,19^ for infections such as CAP, AOM and ABRS, has over time become less susceptible to amoxicillin/clavulanic acid in some countries^20^ since the MICs of some isolates have increased. When treating infections, it is important to be able to eradicate bacterial pathogens with raised MICs to optimize clinical outcome while at the same time minimizing the risk of selecting variants with even higher MICs. This is possible because β-lactams, unlike many other antibiotics, have time-dependent killing properties. Their efficacy depends on the amount of time the drug concentration is present at the site of action. Although increasing the concentration at the infection site above a particular concentration will not have any effect on the efficacy, the use of higher doses and/or more-frequent dosing allows for successful eradication of infections caused by pathogens with higher MICs because the time above the MIC is increased.^21^

#### ATLAS

**Figure 1.**
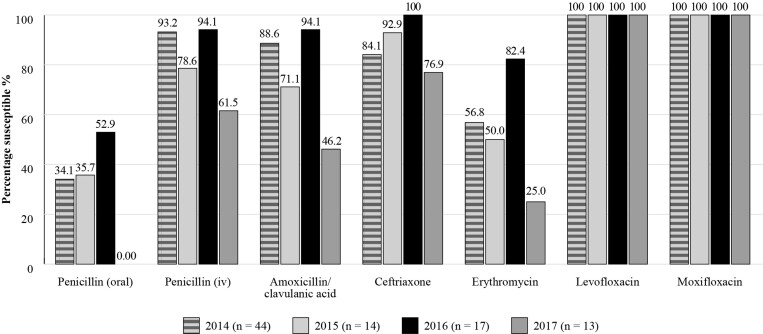
Percentage susceptibility rates based on CLSI breakpoints for antibiotics against *S. pneumoniae* isolates from the ATLAS surveillance programme in Mexico 2014–17. Data access date 21 November 2021.

**Figure 2.**
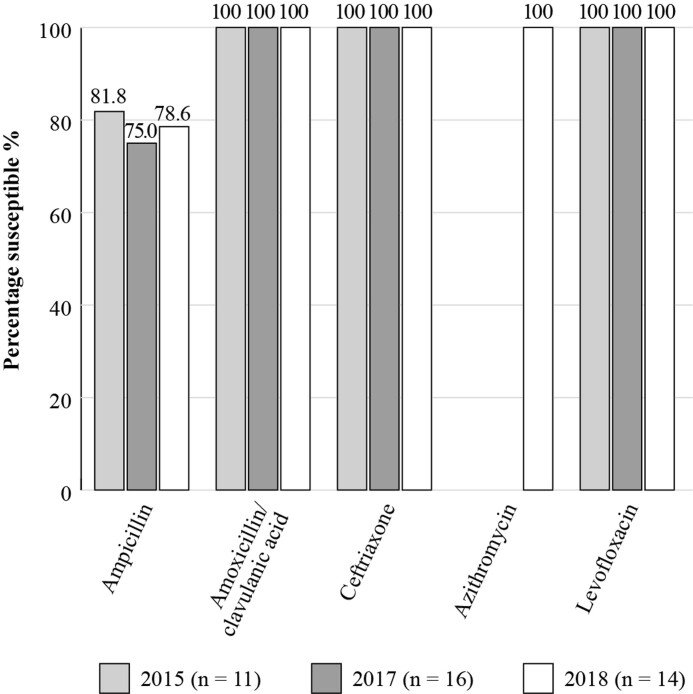
Percentage susceptibility rates based on CLSI breakpoints for antibiotics against *H. influenzae* isolates from the ATLAS surveillance programme in Mexico 2015–18. Data access date 21 November 2021.

The Antimicrobial Testing Leadership and Surveillance (ATLAS) database is a global AMR surveillance programme which is fully searchable, available for general access and records the susceptibilities of a range of bacterial and fungal pathogens to a bank of antimicrobials.^22^ Within ATLAS, susceptibility data for Mexico are available for a relatively small number of isolates. Figure 1 shows the susceptibility based on CLSI breakpoints of *S. pneumoniae* isolates collected in 2014 to 2017. The susceptibility of *S. pneumoniae* to the antibiotics tested decreased overall during this period, apart from the fluoroquinolones, with isolates remaining fully susceptible to both levofloxacin and moxifloxacin. Results are available on the ATLAS database for *H. influenzae* isolates collected in 2015, 2017 and 2018 and are shown in Figure 2.

#### SENTRY

**Figure 3.**
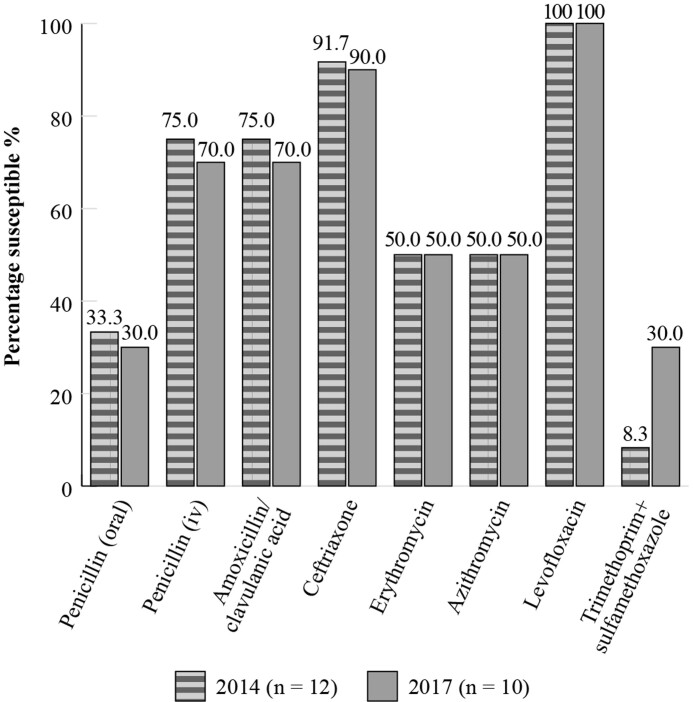
Percentage susceptibility rates based on CLSI breakpoints for antibiotics against *S. pneumoniae* isolates from the SENTRY surveillance programme in Mexico 2014–17. Data access date 21 November 2021.

**Figure 4.**
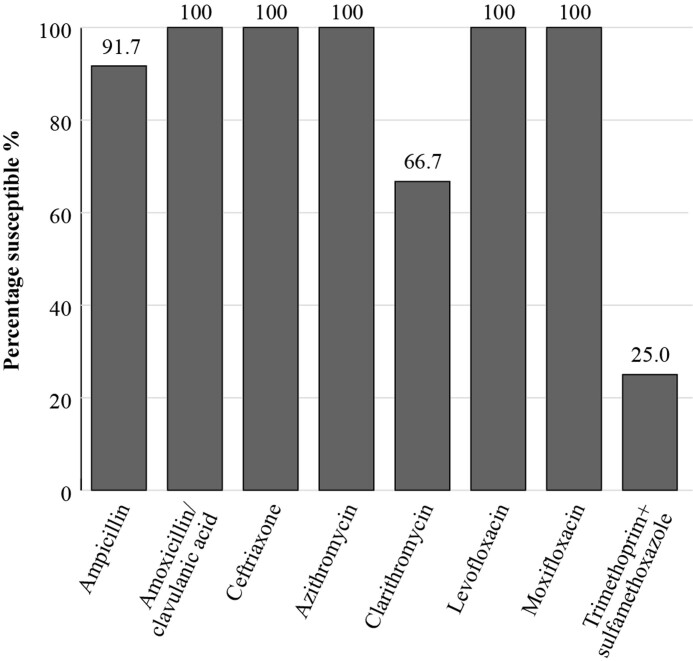
Percentage susceptibility rates based on CLSI breakpoints for antibiotics against *H. influenzae* isolates (*n* = 12) from the SENTRY surveillance programme in Mexico 2018. Data access date 21 November 2021.

The SENTRY Antimicrobial Surveillance Programme was initiated in January 1997 and was designed to monitor the predominant pathogens and AMR for nosocomial and community-acquired infections globally. Data are available for susceptibility of *S. pneumoniae* and *H. influenzae* isolates collected in Mexico although isolate numbers are again relatively low.^23^ The susceptibility rates, by CLSI breakpoints, from SENTRY shown in Figures 3 and 4 confirm the susceptibility picture presented by the ATLAS results for Mexico.

#### GLASS

In 2015, the WHO launched the Global Antimicrobial Resistance and Use Surveillance System (GLASS). GLASS is a global system that collects national AMR data for selected bacterial pathogens that cause common infections. The aim is to monitor the prevalence of AMR among major pathogens in clinical settings^24^ to provide the supporting data required to ensure that countries can design cost-effective, evidence-based AMR response strategies. During the first four years, 91 countries or territories have enrolled in GLASS and data for over two million patients from 66 countries are included.^25^ Pathogens currently included in GLASS-AMR are: *Acinetobacter* spp., *E. coli, Klebsiella pneumoniae*, *Neisseria gonorrhoeae*, *Salmonella* spp., *Shigella* spp., *S. aureus*, and *S. pneumoniae* and a new and important component is the inclusion of antimicrobial consumption (AMC) surveillance at the national level.^26^

Whilst Mexico is not currently one of the countries that has enrolled in GLASS, the WHO Regional Office for the Americas/Pan American Health Organization supports AMR surveillance, prevention and control in the Region through its AMR special programme and Mexico is one of the countries included in that initiative.

## Disease Management Guidelines

For management of the common CA-RTIs, CAP, AOM and ABRS in Mexico, clinicians use several country-specific local antibiotic prescribing guidelines plus a range of international guidelines, examples of which are included in Table 1. Most guidelines suggest a first-line antibiotic or antibiotics along with an alternative or alternatives and then a second-line antibiotic or antibiotics, also with an alternative or alternatives. The first-line antibiotic is the recommended first choice that should be prescribed by the clinician following diagnosis of the infection, supported by the criteria defined by the organization or committee; alternatives may be provided for use in particular circumstances, for example, if the first-line antibiotic is a β-lactam antibiotic, then alternative suggestions will be for use in the case of penicillin allergy. The second-line antibiotic is for use if the first-line antibiotic does not achieve the anticipated outcome, and again alternatives may be included for use under specific circumstances.

**Table 1.** Examples of local and international antibiotic prescribing guidelines referred to by physicians in Mexico for the management of community-acquired respiratory tract infections

Local antibiotic prescribing guidelines
Diagnosis and Treatment of Community-acquired Pneumonia in Patients from 3 months to 18 years in the First and Second Level of Care, Update 2015^27^
Acute Rhinosinusitis in Pediatric Age 2015^29^
Prevention, Diagnosis and Treatment of Community-acquired Pneumonia, Update 2017^28^
Prevention, Diagnosis and AOM Treatment in Paediatric Age 2021^30^
International antibiotic prescribing guidelines
IDSA 2007: Infectious Diseases Society of America. Guidelines on the Management of Community-acquired Pneumonia in Adults^31^
IDSA 2011 (Endorsed by AAP): The Management of Community-acquired Pneumonia in Infants and Children Older than 3 Months of Age: Clinical Practice Guidelines by the Pediatric Infectious Diseases Society and the Infectious Diseases Society of America^32^
IDSA 2012: IDSA Clinical Practice Guideline for Acute Bacterial Rhinosinusitis in Children and Adults^33^
AAP 2013: American Academy of Pediatrics. The Diagnosis and Management of Acute Otitis Media^34^
IDSA 2019: Diagnosis and Treatment of Adults with Community-acquired Pneumonia. An Official Clinical Practice Guideline of the American Thoracic Society and Infectious Diseases Society of America^35^
EPOS 2020: European position paper on rhinosinusitis and nasal polyps 2020^36^

### International antibiotic prescribing guidelines

For the management of CAP in adults and paediatrics, the international guidelines referred to by clinicians in Mexico include those from the IDSA.^32,35^ For example, the first-line recommendation by the IDSA 2019 for treating adult outpatients with CAP^35^ is amoxicillin or doxycycline or a macrolide (with no comorbidities/or risk factors for MRSA or *P. aeruginosa*/if local pneumococcal resistance is <25%) but if the patient has comorbidities and CAP, the recommendations include amoxicillin/clavulanic acid, 500 mg/125 mg given three times daily, 875 mg/125 mg or 2000 mg/125 mg both given twice daily, or cephalosporins in combination with a macrolide or doxycycline, or monotherapy with a respiratory fluoroquinolone.^35^

For the management of AOM, the international guidelines used in Mexico include those from the American Academy of Pediatrics (AAP)^34^ and for ABRS in adults and paediatrics the international guidelines referred to in Mexico include those from the IDSA.^33^ For ABRS in adults the IDSA recommend first-line amoxicillin/clavulanic acid 500 mg/125 mg three times daily or 875 mg/125 mg twice daily.^33^

### National antibiotic prescribing guidelines

For the management of CAP, AOM and ABRS, the commonly used national guidelines, which are mainly used in public institutions are, firstly for the treatment of CAP ‘Diagnosis and treatment of CAP in patients from 3 months to 18 years in the first and second level of care’, update 2015,^27^ in which amoxicillin 80–90 mg/kg/day given in two doses up to a maximum of 4 g per day is recommended as a first-line treatment and amoxicillin/clavulanic acid 90 mg/kg/day given in two doses up to a maximum of 4 g per day, is recommended as an alternative treatment for outpatients with bacterial CAP. In cases of penicillin allergy, azithromycin, clarithromycin or erythromycin are the recommendations. The prevention, diagnosis and treatment of CAP, update 2017^28^ also recommends amoxicillin as a first-line, and amoxicillin/clavulanic acid as an alternative treatment in adults with low-risk CAP. For AOM, the national treatment guideline for prevention, diagnosis and treatment in paediatric patients recommends, for example, amoxicillin 80–90 mg/kg/day for 5–10 days in children with no previous antibiotic treatment, and amoxicillin/clavulanic acid 90 mg/kg/day for 5–10 days for children who have had previous antibiotic treatment.^30^

## Antibiotic availability

Access to antibiotics may be an issue for patients in low- and middle-income countries due to cost and insufficient government expenditure or support in this area. Drug supply chains may also contribute to the problem. Limited access to the most appropriate antibiotic to treat a specific infection may result in raised mortality from treatable bacterial infections, and the use of suboptimal amounts of antibiotic facilitates resistance development and allows resistant strains to persist.^37,38^

Antibiotic availability within a country is an important consideration when applying recommendations from guidelines. In Mexico, currently available formulations of amoxicillin/clavulanic acid are mentioned as first- or second-line recommendations by several of the RTI antibiotic prescribing guidelines. In particular, amoxicillin/clavulanic acid 90 mg/kg/day (high dose) is available in Mexico and is recommended by IDSA 2007 CAP treatment guidelines^31^ for outpatients with comorbidities, by the IDSA 2011 guidelines for the treatment of paediatric CAP in outpatients,^32^ by the IDSA 2012 treatment guidelines for sinusitis^33^ as initial treatment for children and in adults with risk of treatment failure, by the AAP 2013 AOM^34^ guidelines as initial or delayed treatment of AOM in children or after failure of initial treatment, and by the IDSA 2019 guidelines for adults with CAP^35^ who have comorbidities. The majority of the antibiotics recommended by CA-RTI guidelines are available in Mexico, e.g. most cephalosporins and macrolides. Generic antibiotics also play an important role.

Substandard poor-quality or falsified antibiotics promote AMR and the spread of drug-resistant infections.^39^ Since poor-quality antibiotics are unlikely to contain the full dose needed to eliminate all of the infecting pathogens, this will encourage resistance to develop and allow resistant strains to survive and be transmitted.^40^

The quality of medicines, specifically antibiotics, is an important consideration in Mexico. The WHO launched a Global Surveillance and Monitoring System (GSMS) for substandard and falsified products.^40^ The GSMS aims to work with WHO member states to improve the quality of reporting of substandard and falsified medical products, and, importantly, to ensure the data collected are analysed and used to influence policy, procedures, and processes to protect public health, at the national, regional and the global level. Use of substandard or falsified antibiotics not only compromises clinical outcomes but also risks increased AMR. The most recent summary (2013–17) reported substandard and falsified medicines in 46 member states, including Mexico, and antibiotics represent 16.9% of all products reported, second only to malaria drugs (19.6%).^40^

## Local insight

### Clinician expert comment

The information included here will have an important relevance to the clinical practice of Mexican physicians because it raises awareness amongst physicians of AMR in common CA-RTI bacterial pathogens. The rational and conscious use of antibiotics by physicians will contribute in a significant way to the decrease of antibiotic resistance in Mexico.

As mentioned previously, in order to tackle AMR, the Mexican government established a law making the sale of antibiotics possible only with a medical prescription. Unfortunately, the impact of this law has not been immediately obvious, however, in some hospitals antibiotic prescribing is controlled by an infectious diseases committee that applies strict criteria for the prescription and indication of antibiotics; this and other measures have decreased the antibiotic resistance of hospital-acquired infections.

When considering the use of an antibiotic, the decision should be made by first considering the local and international surveillance data, but unfortunately in Mexico there is limited information regarding this topic. In terms of upper airway infections, the information is provided by the GIVEBPVac data related to invasive strains from 2019.^14^ Data provided by the IDSA guidelines, AAP guidelines for acute otitis media and rhinosinusitis management, the AAO-HNS guidelines and the Pan-American clinical practice guideline for medical management of acute tonsillitis and adenoids hypertrophy could also be taken into account. To summarize, there is insufficient information in Mexico about AMR in pathogens causing upper airway infections. Guidelines are of benefit to the clinical practice, especially if they include local information.

## Conclusions

In an era of rising AMR throughout the world, this paper aims to define areas where action is required to tackle AMR by analysing and understanding the current situation within Mexico. Information is presented concerning antibiotic use and prescribing, approach to AMR, availability of local susceptibility data, use of international and/or local management guidelines and how these link to antibiotic availability. To our knowledge this is the first time this information has been reviewed and presented in detail for a specific country.

Inappropriate antibiotic prescribing in Mexico has been widespread and associated with antibiotic resistance. Historically, antibiotic usage in Mexico has been high, in fact in 1997 Mexico was the leading country in Latin America for antibiotic clinical use. However, unlike most of the countries throughout the world, the antibiotic consumption rate in Mexico fell between 2000 and 2015 by between 10 and 15 DDDs per 1000 inhabitants per day. A recent reduction could also be a reflection of the law banning the OTC sale of antibiotics, although, as mentioned in the clinician’s expert comment, the effect of this law may not have been felt immediately. Individual hospitals in Mexico may have their specific regulations concerning antibiotic use but similar restrictions relating to community medicine are not so obvious.

In terms of surveillance, there is a lack of a national network for measuring antibiotic resistance; available data comprises local studies only. Currently, results from the global surveillance studies ATLAS and SENTRY on the susceptibility of CA-RTIs reveal some lower levels of susceptibility amongst the common respiratory pathogens for some antibiotic classes, such as the macrolides. The fluoroquinolone antibiotics have so far retained activity, although as mentioned in the introductory paper to this Supplement,^2^ regulatory bodies and other authors urge caution, restricting their use to limited situations due to serious safety concerns.^41–44^

The Mexican clinician’s expert view is that more local susceptibility information is required. Surveillance studies are performed but they are either part of global studies and isolate numbers are relatively small, or local Mexican studies, such as GIVEBPVac data, only relate to invasive strains. While a range of international and local guidelines are utilized by clinicians in Mexico, a more standardized inclusive approach is needed to develop up-to-date local country-specific guidelines for CA-RTI management. These guidelines would be based on recent surveillance data of isolates from community-acquired infections which would make them more locally relevant for clinicians, reiterating the Consensus Principles as described in the introductory paper to this Supplement.^2^ This would pave the way for improved adherence and a higher level of appropriate antibiotic prescribing in CA-RTIs which could, in turn, potentially limit AMR development and improve clinical outcomes for patients in Mexico.
